# Development of the Precision Link Biobank at Boston Children’s Hospital: Challenges and Opportunities

**DOI:** 10.3390/jpm7040021

**Published:** 2017-12-15

**Authors:** Florence T. Bourgeois, Paul Avillach, Sek Won Kong, Michelle M. Heinz, Tram A. Tran, Ramkrishna Chakrabarty, Jonathan Bickel, Piotr Sliz, Erin M. Borglund, Susan Kornetsky, Kenneth D. Mandl

**Affiliations:** 1 Computational Health Informatics Program (CHIP), Boston Children’s Hospital, Boston, MA 02115, USA; Paul_Avillach@hms.harvard.edu (P.A.); SekWon.Kong@childrens.harvard.edu (S.W.K.); Michelle.Heinz@childrens.harvard.edu (M.M.H.); Jonathan.Bickel@childrens.harvard.edu (J.B.); Piotr.Sliz@childrens.harvard.edu (P.S.); Erin.Borglund@childrens.harvard.edu (E.M.B.); Kenneth.Mandl@childrens.harvard.edu (K.D.M.); 2 Division of Emergency Medicine, Boston Children’s Hospital, Boston, MA 02115, USA; 3 Department of Pediatrics, Harvard Medical School, Boston, MA 02115, USA; 4 Department of Biomedical Informatics, Harvard Medical School, Boston, MA 02115, USA; 5 Division of Endocrinology, Boston Children’s Hospital, Boston, MA 02115, USA; 6 Biobank Core Lab, Biorepository, Boston Children’s Hospital, Boston, MA 02115, USA; Tram.Tran@childrens.harvard.edu; 7 Information Services Department, Business Intelligence & Clinical Research Informatics, Boston Children’s Hospital, Boston, MA 02115, USA; Ramkrishna.Chakrabarty@childrens.harvard.edu; 8 Department of Biological Chemistry & Molecular Pharmacology, Boston, MA 02115, USA; 9 Office of Clinical Investigations, Boston Children’s Hospital, Boston, MA 02115, USA; Susan.Kornetsky@childrens.harvard.edu

**Keywords:** pediatric biobank, biospecimens, biorepository

## Abstract

Increasingly, biobanks are being developed to support organized collections of biological specimens and associated clinical information on broadly consented, diverse patient populations. We describe the implementation of a pediatric biobank, comprised of a fully-informed patient cohort linking specimens to phenotypic data derived from electronic health records (EHR). The Biobank was launched after multiple stakeholders’ input and implemented initially in a pilot phase before hospital-wide expansion in 2016. In-person informed consent is obtained from all participants enrolling in the Biobank and provides permission to: (1) access EHR data for research; (2) collect and use residual specimens produced as by-products of routine care; and (3) share de-identified data and specimens outside of the institution. Participants are recruited throughout the hospital, across diverse clinical settings. We have enrolled 4900 patients to date, and 41% of these have an associated blood sample for DNA processing. Current efforts are focused on aligning the Biobank with other ongoing research efforts at our institution and extending our electronic consenting system to support remote enrollment. A number of pediatric-specific challenges and opportunities is reviewed, including the need to re-consent patients when they reach 18 years of age, the ability to enroll family members accompanying patients and alignment with disease-specific research efforts at our institution and other pediatric centers to increase cohort sizes, particularly for rare diseases.

## 1. Introduction

The last two decades have seen a remarkable increase in the establishment of biobanks, especially those facilitating a broad research agenda across diseases, treatments and outcomes [1]. The mission and organization of these biobanks vary widely in numbers and types of specimens stored, patient populations targeted and the availability of linked clinical data from electronic medical records [1,2]. What many share, however, is the potential to advance our understanding of the genetic basis of disease and to support translational research studies and the development of diagnostic and therapeutic approaches personalized to individual patients, including children [3,4].

Pediatric-focused biobanks represent a particularly rich opportunity, but they face unique challenges including rarity of conditions and logistical and regulatory barriers to recruitment of children into research studies [5,6]. Large pediatric study populations can be efficiently assembled through biobanks and used to study rare diseases, pediatric-specific disease markers and responses to treatment and longitudinal clinical phenotypes. However, most United States (U.S.) biobanks enroll exclusively adult participants, with only 44% including any children and 2% focused entirely on pediatric patients [1]. A number of pediatric biobanks has been established, including at Boston Children’s Hospital. In an effort to further inform the advancement of pediatric biobanks and provide guidance to other institutions developing this resource, we describe here our institutional biobank, including basic organizational features, challenges and opportunities identified and comparison to a sampling of other U.S.-based pediatric biobanks.

## 2. Development and Launch

The Precision Link Biobank at Boston Children’s Hospital (“Biobank”) was established after extensive consultation and close partnership with a number of stakeholders at our institution. Input was solicited from investigators, the Institutional Review Board (IRB), research strategy committees, clinical governance committees, division chiefs and a patient family advisory committee, among others. It is structured as a research protocol and was approved by the Institutional Review Board at Boston Children’s Hospital. The Biobank was gradually phased in, operating initially as a pilot project in a limited number of clinical settings with particular interest in patient participation in the Biobank, before launching hospital-wide in 2016. Throughout the pilot phase, all patient encounters were carefully recorded and monitored, including outcomes of conversations with research assistants and reasons for decline. Based on the positive reception by patients, families, clinical staff and investigators, recruitment was expanded to additional clinical settings and enrollment models throughout the hospital.

## 3. Enrollment via Informed Consent

All patients at Boston Children’s Hospital are eligible for enrollment in the Biobank. Research assistants obtain informed consent for all participants, and the consent form is signed by a legal guardian for participants under 18 years of age (Supplementary Materials: informed consent document). Participants who are 13 to 17 years of age are asked to provide assent by also signing the form after a verbal discussion. Particular attention is given to the discussion of how we handle personal health information, or identifiable data, which includes participant name, medical record number, date of birth and certain other dates. The informed consent process is performed using an electronic consenting system administered on tablets that are handed to patients. The electronic system presents a digital version of the consent document, enhanced with an introductory video, pop-up glossary terms and a feature to highlight areas requiring clarification.

The consent gives permission for the following:

- Research use of electronic health record (EHR) data. EHR data available for study include participant demographics, dates and types of encounters (e.g., emergency department (ED) visit, hospitalization), clinic and hospital discharge notes, diagnoses, laboratory orders and values, physical exam findings, medications prescribed and procedures performed. Boston Children’s Hospital researchers may request the use of identifiable data after a separate approval has been obtained from the IRB.
- Research use of residual specimens collected in the process of routine clinical care. This encompasses all bodily fluids and tissues, including blood, plasma, cerebrospinal fluid, urine, fecal samples, buccal swabs, biopsies, aspirates and tissue specimens from any organ that are no longer needed for the clinical care of a patient.
- Sharing of de-identified clinical data and specimens outside of the institution. This may occur in the form of collaborations with investigators at other medical centers, deposits to central National Institute of Health repositories (e.g., the database of Genotypes and Phenotypes (dbGaP)) and partnerships with pharmaceutical companies.
- Ongoing data collection. Any clinical data available in the EHR and specimens available at the time of consent become available to the Biobank. In addition, the Biobank may collect and use additional data and specimens prospectively until the participant turns 18 years old, at which time participants can re-enroll as adults.
- Re-contact for additional data and/or participation in clinical studies. Participants allow the Biobank to contact them to obtain additional health information, to request additional samples and to inform them of eligibility for specific research studies.
- Broad range of research activities. The types of research activities that may be conducted using data and specimens from the Biobank include genetic analyses, creation of immortalized cell lines and pluripotent stem cells, biomarker analyses and epidemiological and outcomes studies. This research may focus on any disease, condition or treatment, regardless of the participant’s clinical history.

In addition, participants are given the opportunity to consent to the provision of a 4-mL blood sample for research use. This sample is not required for enrollment in the Biobank. The sample may be drawn in conjunction with other scheduled clinical tests.

Once a participant turns 18 years of age, she/he must re-consent as an adult to allow for continued data and sample collection. All information collected up to her/his 18th birthday may continue to be used under the original consent unless she/he withdraws from the study. Participants are able to withdraw at any time in which case samples and data are not used for any future research studies, though research on data and specimens distributed to investigators prior to the withdrawal request may continue.

## 4. Recruitment Approaches in Different Clinical Settings

Dedicated research assistants enroll participants in-person in clinical settings throughout the hospital, including outpatient clinics, inpatient units, the ED and general waiting areas, such as in phlebotomy or radiology. Initially, patients were identified prior to clinical visits whenever possible and provided with an informational mailing about the Biobank, but we found that this did not have a substantial impact on knowledge at the time of the encounter with Biobank staff (e.g., few recalled receiving the information), and thus, pre-contact was discontinued. In each of the clinical settings, research assistants work closely with clinical staff to tailor enrollment procedures to the clinic workflow and ensure minimal disruptions in clinical proceedings (e.g., approach patients in the waiting area vs. in the examination rooms). They also coordinate with other research staff recruiting in the clinics in order to ensure that patients are not approached more than once during any clinical encounter.

The consent and enrollment process typically takes less than 15 min and is conducted in the waiting room, clinical examination rooms or, when available, dedicated research enrollment areas. Research assistants explain the key provisions of the Biobank and ask patients and their families or legal guardians to review the written informed consent documents. Our research staff answer any questions that arise and guide participants through the varying levels of participation, including the option to have a dedicated research blood draw. The consent is currently available in four languages: English, Spanish, Portuguese and Arabic. For all non-English participants, the informed consent process is completed with the assistance of a trained hospital interpreter. In addition, hospital interpreter services are available to translate the consent into other additional languages as needed.

Patients can choose to enroll, decline or to defer a decision, in which case they are considered “undecided”. Every interaction and outcome with potential participants is tracked in our research database. Patients who decline may be approached once more no less than six months later and, if they decline again, are not approached again. Patients who are undecided may be approached a total of five times, with no less than seven days between interactions. If they remain undecided, they will no longer be approached, but are free to contact us in the future to enroll or to decline participation.

Certain clinical settings have dedicated research staff that conduct and manage all patient recruitment and enrollment to research studies. In these settings, we provide training to the local research assistants who assume responsibility for enrollment to the Biobank. This offers a number of advantages, as this research staff is most familiar with the optimal approaches to engage with families in their clinics and navigate the clinical workflow. Each of these research personnel is first trained by the Biobank staff and then paired with a Biobank staff member who is available to field questions and manage data coordination with our Biobank systems.

A number of physician investigators have elected to enroll their own patients in order to build a cohort of specific patient populations in the Biobank for future study. Examples include patients with sickle cell anemia and non-alcoholic fatty liver disease. In these cases, a training session is conducted with the physicians to orient them to the details of the Biobank, the consent process and the information that must be collected at the time of enrollment. Research assistants remain in close contact with the physician investigators to facilitate enrollment and manage all participant-tracking procedures.

## 5. Use of “Merged” Consent Forms

In an effort to align the Biobank with other research studies that involve sample collection at our institution and create efficiencies through a centralized, shared resource, we have designed an approach to merge our protocol and consent with existing studies. Through close collaboration with investigators leading disease-specific specimen repositories at Boston Children’s Hospital, we have created “merged” consent forms in which participants consent to enrollment in both the Biobank and the investigator’s specific study through one enrollment encounter and consent form. This merged consent option is available to any investigator interested in leveraging the resources of the biobank for their studies. In this case, participants consent to the standard elements of the Biobank, including research use of health data and discarded specimens, as well as any specific data and sample requirements under the disease-specific protocol. The research blood draw is generally not optional in this case, as it is a required feature of many other repositories, which do not rely on discarded specimens and require the sample for genetic analysis. This sample is collected by the Biobank where it is available to the specific investigator, as well as to other researchers at the institution. Through consent to the specific investigator protocol, additional health information, laboratory and clinical testing and sample collection may be performed, as outlined in the investigator’s protocol. These processes occur independent of the Biobank, though the information and results may be included in the participant’s Biobank record and thereby made available to other researchers at the institution.

## 6. Sample Collection and Management by the Biobank

For participants who consent to the research blood draw, the Biobank collects a 4-mL blood sample collected in an EDTA tube. This sample is collected by phlebotomists or nurses using a research laboratory order that is placed in the participant’s EHR. The samples are sent to our central clinical laboratory from where they are routed to the Biobank for processing and storage. The Biobank can also collect saliva specimens, where participants unable to provide a blood sample may give a saliva sample instead. These samples are delivered or sent directly to the Biobank after collection using saliva collection kits.

Protocols employing a merged consent frequently require other types of research samples, such as blood for serum analysis, stool samples or urine samples for analyses specific to their protocol. The Biobank can place research orders in the EHR for these to be collected and sent to the Biobank by the central clinical laboratory or research staff.

In addition to these research samples, the Biobank may collect all residual clinical specimens for consented patients at the conclusion of the clinical embargo. This embargo period ensures that all clinical work can be completed prior to sample distribution for research purposes. The length of this embargo period varies for different types of samples, ranging from seven days for blood samples to up to one year for tissue specimens. All residual blood samples drawn in EDTA tubes are identified through automated reports run in our EHR system and collected by Biobank staff. Multiple residual samples may be collected until a volume of 10 mL is reached, at which point additional samples are discarded. Similarly, all pathology specimens for consented patients are deposited in the Biobank. At this time, other types of specimens are not routinely collected unless an investigator requests a particular sample type, potentially in a specific patient population, in which case these specimens are also tagged and become routinely collected. For example, cerebrospinal fluid obtained from patients in the intensive care unit or urine specimens from patients evaluated for appendicitis in the ED might be specifically identified, collected and stored.

Specimens are stored and managed by the Biobank Core Lab facility at Boston Children’s Hospital. This facility handles specimens for a number of different projects and investigators and consists of 2400 square feet of wet lab space and a freezer farm. Each specimen submitted to the Biobank is verified upon receipt, entered into our laboratory database and assigned a unique ID for accurate sample tracking and management. The database stores key sample information, including sample history and chain of custody, as well as phenotypic data and patient demographics. Each freezer is protected with emergency power, redundant alarm systems and CO_2_ backup systems.

Biobank specimens are tracked and available for de-identified browsing using a customized version of the STARLIMS laboratory information management system [7]. In addition to standard sample metadata, such as data on collection date and quantity, we store specimen-specific metadata for nucleic acid samples, cells and tissue specimens. The software interfaces with other institutional informatics systems, including the hospital EHR, to reduce data duplication and maintain specimen, patient and collection data integrity across systems. Project role-based permission settings are designed to control access to different features and data.

**Figure 1:**
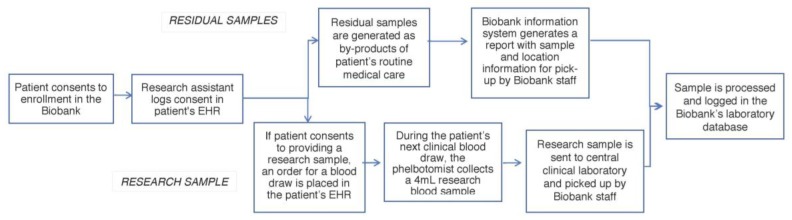
Biobank sample workflow. Residual samples are biospecimens that are typically discarded after clinical procedures (e.g., blood, tissue, urine). These are collected from all patients enrolled in the Biobank. A research sample is a biospecimen collected for research purposes. Participants are given the option to agree to this additional sample during the consent process. EHR: electronic health record.

Research and residual clinical sample collection are integrated into the institutional clinical and research informatics infrastructure (Figure 1). Research assistants place a laboratory order in the EHR of consented patients for the sample to be collected. This order triggers a daily notification for sample pick-up and transport from the clinical laboratory to the Biobank core facility. For residual clinical samples, our Biobank information system scans the EHR of consented patients and generates a report with sample and location information for pick-up. Upon pick-up, the whole blood is frozen at −80 °C. Samples are stored indefinitely or until they are released to approved researchers. While not yet our standard operating procedure, trial runs have indicated that an average of 8 µg can be extracted from 0.5 mL of whole blood.

## 7. Return of Research Results and Patient Privacy

During the consent process, participants are informed that individualized return of results is not part of the standard procedures of the Biobank. Similarly, results from studies are not added to the patient medical record or communicated to their physicians. Participants will not be notified if and/or when their samples are used in a study. Additionally, they are informed that neither they nor their physician should expect individual results from any study. Participants are made aware that if there is a result that is determined to be of sufficient medical importance to their child or themselves, Biobank staff, in consultation with the IRB, may contact the participant directly or through the participants’ physician, to counsel them about possible result return. Risks of genetic research and issues of privacy are also reviewed, including protections in place at the federal and institutional level (e.g., Certificate of Confidentiality) to maintain patient confidentiality and prevent discrimination based on genetic information.

## 8. Data Integration, Sample Access and Distribution to Investigators

Biobank consent status, demographic details collected as part of the enrollment process and basic Biobank specimen characteristics are complemented with phenotypic data automatically derived from the hospital’s EHR data warehouse, including International Classification of Diseases, Ninth Revision (ICD-9) encoded diagnoses, laboratory orders and results, radiology orders, procedures performed, medications administered and prescribed and types of encounters. Data are stored in an instance of the i2b2/TranSMART open source data analytic platform hosted on Amazon Web Service (AWS) [8,9]. A Business Associate Agreement between AWS and Boston Children’s Hospital ensures full compliance with the Health Insurance Portability and Accountability Act. For specimens undergoing whole exome sequencing, variant call format and Binary Alignment Map (BAM) format files are stored in i2b2/TranSMART, as well.

Investigators are able to search the Biobank inventory through feasibility queries performed on de-identified participant data. These searches are performed using i2b2/tranSMART, which enables investigators to independently identify specific research studies the Biobank can support [10]. For example, an investigator can determine how many participants have been enrolled who have a diagnosis of asthma, are six years or older, have had at least one hospitalization and have a DNA sample available in the Biobank. 

Use of samples and data is available and free of charge to all Boston Children’s Hospital investigators. Only investigators based at Boston Children’s Hospital are able to be the primary requestors of Biobank data and samples. All requests require approval by the Sample and Data Access Committee, an institutional committee with representation across departments and disciplines. This entity is charged with reviewing, prioritizing, approving and coordinating requests for use of the Biobank infrastructure, samples, phenotype data and patient contact options.

Investigators may apply for de-identified or identifiable data, depending on the scientific design of their study. For de-identified data, investigators apply directly to the Biobank, which acts as an ‘Honest Broker’ [11]. In this case, the Biobank holds the key to the identifiable data, but distributes only de-identified data and samples to the investigator. If identifiable data are needed (e.g., medical record number for more detailed phenotype extraction from clinical notes), an IRB-approved protocol is required that justifies the need for use of personal health information. To date, six requests have been approved providing data on 225 participants and samples from 69 participants.

Investigators sign a data use agreement prior to sample and data release that outlines the terms of use and access to the samples and data (Supplementary Materials: data use agreement form). The recipient investigator agrees, among other terms, to use the data and samples as outlined in the proposal, to not share the data and samples with any entities beyond what has been set forth in the proposal and, if identifiable information is to be released, to maintain all participant information as confidential. In addition, the investigator agrees to inform the Biobank of any sample derivatives at the completion of the project, including extracted DNA, cell lines and genetic data files, and to provide these to the Biobank for future research use, if requested.

Investigators may collaborate with outside entities and share de-identified samples and data. These collaborations must be outlined in the request submitted to the Biobank Sample and Data Access Committee and may include partnerships with other academic centers, with research studies based in for-profit organizations, or with other repositories and research networks.

## 9. Current Enrollment Status and Participant Characteristics

Since the launch of the Biobank, 4900 patients have enrolled, of which 2750 (56.1%) agreed to provide a research blood specimen. Currently, we are enrolling over 225 patients per month. Table 1 provides basic demographic and clinical information on these patients.

For a total of 2009 (41%) participants, we have collected associated blood samples for DNA processing. Of these, 896 (44.6%) are from research blood samples and 1113 (55.4%) represent residual blood samples of at least 1 mL. In addition, a total of 221 (4.5%) participants also have tissue specimens stored in our Biobank.

## 10. Pediatric-Specific Considerations: Challenges and Opportunities

Several aspects of the enrollment and sample collection process are pediatric-specific. The informed consent is conducted with parents or legal guardians, though the child may also participate at various time points. During the enrollment process, participants 13 to 17 years of age are included in the informed consenting process and asked to provide assent and sign the consent form along with their parent or legal guardian. When participants reach maturity at 18 years of age, they must be re-consented to allow ongoing sample collection and access to EHR data recorded after their 18th birthday. Research on samples and data that have already been distributed may continue. While the re-consenting process can be challenging as contact information for the participant is not always available, this process affords the opportunity to re-engage participants, inform them of ongoing research projects and new ones for which they might be eligible and to collect additional clinical data.

Another unique opportunity emerges in the process of consenting participants through their parents: we are able to recruit parents and other family members to the Biobank along with the child. Analyzing sequencing data from trios—the child and her/his biologic parents—enhances capacity to identify relevant variants, particularly in regions that are not well covered by sequencing platforms, and the ability to directly observe the inheritance of these variants.

Enrolling sufficiently large cohorts of pediatric patients can be challenging because of the small number of patients with certain diseases or other specific features. We have taken several approaches to increase our recruitment and enrollment volume. First, we have aligned our Biobank with disease-specific research efforts at our institution such that patients may enroll in these disease-specific studies, while also contributing data to a centralized Biobank. This enables us to expand the enrollment of certain patient populations and allow their samples and data to be accessed by investigators across the institution. In addition, we are actively engaged in building a network of pediatric institutions that will share de-identified sample and clinical data from participants in their respective biobanks [12]. This type of data pooling holds tremendous potential to augment the study of patients with rare diseases. Finally, in an effort to directly facilitate the enrollment process, we have launched an electronic consenting system in which participants are able to perform the consenting process independently using handheld tablets during their clinical visit [13]. Research assistants can engage with multiple families simultaneously with this approach and remain available to provide assistance and answer questions. We are currently expanding the electronic consenting platform to support remote consenting, by which participants can complete the enrollment process without a clinical encounter [14].

Research-specific blood samples are optional in our Biobank as parents may be concerned about having additional blood drawn during a scheduled clinical test. As a result, we do not have research blood specimens on all participants. In addition, even among those who do consent to the research blood draw, pediatric participants typically only provide the research specimen when clinical testing is required, and this may not occur at every encounter. In order to supplement the research samples, our Biobank has implemented a tracking system to systematically identify all residual blood specimens for collection. This system has allowed us to increase the percentage of patients with a viable specimen for DNA sequencing from 15% to 41%. In addition, it allows us to collect other types of clinical specimens (e.g., residual biopsy tissue) beyond the research sample.

As an additional approach to increasing specimen data, we are targeting patients for Biobank enrollment who are having genetic testing (e.g., whole exome sequencing) performed as part of their clinical evaluation. These patients have sequencing data deposited in their EHR, and Biobank investigators can access these data through approved protocols. For these participants, research blood samples are not necessary.

## 11. Discussion/Conclusions

Table 2 illustrates selected U.S.-based pediatric biobanks to highlight different approaches in mission and operational features. While several enroll all eligible participants, some focus on specific conditions and patient populations. When informed consent is obtained, dedicated research samples—including blood, urine, saliva, CSF and tissue—are collected, sometimes in addition to residual clinical samples. All biobanks link at least some clinical data to the samples, though not all include personal identifiers. Different types of re-contact of participants are supported, including informing participants of other potential research studies of interest and receiving information on incidental research findings. All biobanks in this sample also support sharing of samples and data with outside investigators in collaborative efforts and are able to prospectively collect data beyond the consent date.

We continue to advance the Biobank with new capabilities including computable phenotyping, electronic consenting and inclusion of additional data types, such as imaging and patient-reported data. Implementation of an electronic consenting system will support both in-hospital and remote participant enrollment extending our reach to diverse patient groups that may not be fully represented in our clinics and inpatient units. In addition, we are expanding our hospital patient portal to include research functionality for patient contact, data collection and notification of research results.

More than 95% of hospitals have adopted EHRs and increasingly, longitudinal data are becoming available to support deep phenotyping [15]. These health system data are also more commonly being complemented by patient reports [16,17]. Hence, there is greater motivation than ever to develop robust processes to collect broadly consented samples linked to phenotype and available for genomic and gene expression testing. Accumulating omics-phenotype databases will drive not only discovery, but also transform care through better characterization of our patients and improved diagnostics [18]. Our efforts are intended to ensure that children benefit from these advances.

## Figures and Tables

**Table 1:** Demographic and clinical information of biobank participants (*N* = 4900) *.

**Mean Age, Years (95% CI)**	12.1	(11.8–12.3)
**Age Groups, Years**		
0–1	352	(7.2%)
2–5	1073	(21.9%)
6–11	1182	(24.1%)
12–17	1339	(27.3%)
18+	954	(19.5%)
**Gender**		
Female	2497	(50.9%)
Male	2395	(48.8%)
Unknown	8	(0.2%)
**Race**		
White	3674	(75.0%)
Black	472	(9.6%)
Asian	168	(3.4%)
Native American/Alaskan Native	9	(0.2%)
Native Hawaiian/Pacific Islander	4	(0.1%)
Other	213	(4.3%)
Prefer not to answer	51	(1.0%)
Unknown	309	(6.3%)
**Ethnicity**		
Non-Hispanic/Latino	3359	(68.6%)
Hispanic/Latino	624	(12.7%)
Prefer not to answer	555	(11.3%)
Unknown	362	(7.4%)
**Participants with at least one clinical visit within the past year**	4437	(91.0%)
Surgical encounter	1210	(24.7%)
Admission to Inpatient unit	1099	(22.5%)
Admission to intensive care unit	362	(7.4%)
**Most Frequented Clinics (Top 10)**		
Pre-op Admitting	2650	(54.1%)
Emergency Department	2276	(46.4%)
Outpatient Labs (Phlebotomy)	1968	(40.2%)
Noninvasive Cardiology	1576	(32.2%)
Radiology/Interventional Radiology	1295	(26.4%)
Gastroenterology	1269	(25.9%)
Orthopedics	1217	(24.8%)
Otolaryngology	1153	(23.5%)
Hearing/Speech	870	(17.8%)
Endocrine	865	(17.7%)

* All values represent frequencies and percentages unless otherwise noted.

**Table 2:** Features of selected U.S.-based pediatric biobanks.

Name	Institution	Patient Population	Consent Type	Sample Types	Medical Record Data Linked to Sample?	Identified Data?	Re-Contact Type	Able to Share Data and/or Samples Outside of Institution?	Prospective Sample and/or Clinical Data Collected (i.e., beyond Consent Date)?
Precision Link Biobank	Boston Children’s Hospital	All	Opt-in, fully-informed consent Any research question	Research blood and saliva samples Residual clinical samples	Yes	Yes	To receive information about the Biobank To receive requests for additional data and samples To learn about potential research studies of interest To receive information on incidental research findings	Yes, de-identified data with local faculty member as co-investigator	Yes
Better Outcomes for Children [19]	Cincinnati Children’s Hospital Medical Center	All	Opt-in, using condensed consent document presented by hospital registrar	Residual clinical samples	Yes, through linkage with a separate repository with clinical data	No	To receive information on incidental research findings	Yes, de-identified data with local faculty member as co-investigator	Yes
Pediatric BioVU [20]	Vanderbilt University Medical Center	All	Opt-out * Any research question related to health and wellness	Residual clinical samples	Yes	No	None	Yes, de-identified data with local faculty member as co-investigator	Yes
Pediatric CNS Biorepository	Children’s National Health System	Patients with neurological diseases	● Different types of consents: - opt-in, fully-informed consent - waived consent - post-mortem consent ● Any research question related to neurological diseases	Research blood, saliva, tissue, CSF, and urine samples Residual clinical samples	Yes, depending on the type of consent obtained	Yes, depending on the type of consent obtained	● Depending on type of consent: - To receive requests for additional data and samples - To request to use the data and samples for other research studies	Yes, de-identified data	Yes
Pediatric Cancer Genome Project	St. Jude’s Children’s Research Hospital	Patients with oncologic and hematologic conditions	Opt-in, fully-informed consent Any research question	Research blood draws and urine samples Residual clinical samples	Yes	Yes	To receive information about the biobank To receive requests for additional data and samples To learn about potential research studies of interest To receive information on incidental research findings	Yes, de-identified data	Yes
The Down Syndrome Achieves Down Syndrome Biobank	Nationwide Children’s **	Patients with Down syndrome	Opt-in, fully-informed consent	Research blood samples	Yes, annotated with specific clinical data	Yes, to the collecting site; de-identified for external sharing	To receive requests for additional data and samples To learn about potential research studies of interest To receive information on incidental research findings	No, for the time being limited to members within network	No
Children’s Brain Tissue Research Consortium	Children’s Hospital of Philadelphia **	Patients with CNS tumors	Opt-in, fully-informed consent Consent waiver for deceased patients	Research saliva samples Residual clinical tissue, blood, and CSF samples	Yes, annotated with specific clinical data	Yes, to the collecting site; de-identified for external sharing	None	Yes, de-identified data	Yes

* Patients must acknowledge that they are choosing not to opt out; ** institution acts as the coordinating center and manages data and sample storage.

## Supplementary Materials

The following Supplementary Material are available online at http://www.mdpi.com/2075-4426/7/4/21/s1.

## Abbreviations

IRB	institutional review board
EHR	electronic health record
ED	emergency department
AWS	Amazon Web Service

## References

[1] Henderson G.E., Cadigan R.J., Edwards T.P., Conlon I., Nelson A.G., Evans J.P., Davis A.M., Zimmer C., Weiner B.J. (2013). Characterizing biobank organizations in the U.S.: Results from a national survey. Genome Med..

[2] Vaught J., Kelly A., Hewitt R. (2009). A review of international biobanks and networks: Success factors and key benchmarks. Biopreserv. Biobank..

[3] Bowdin S., Gilbert A., Bedoukian E., Carew C., Adam M.P., Belmont J., Bernhardt B., Biesecker L., Bjornsson H.T., Blitzer M. (2016). Recommendations for the integration of genomics into clinical practice. Genet. Med..

[4] Schwartz M.L., Williams M.S., Murray M.F. (2017). Adding protective genetic variants to clinical reporting of genomic screening results: restoring balance. J. Am. Med. Assoc..

[5] Steinbrook R. (2002). Testing medications in children. N. Engl. J. Med..

[6] Bourgeois F.T., Murthy S., Pinto C., Olson K.L., Ioannidis J.P., Mandl K.D. (2012). Pediatric versus adult drug trials for conditions with high pediatric disease burden. Pediatrics.

[7] STARLIMS. Computer Software.

[8] Scheufele E., Aronzon D., Coopersmith R., McDuffie M.T., Kapoor M., Uhrich C.A., Avitabile J.E., Liu J., Housman D., Palchuk M.B. (2014). tranSMART: An open source knowledge management and high content data analytics platform. AMIA Jt. Summits Transl. Sci. Proc..

[9] Murphy S.N., Weber G., Mendis M., Gainer V., Chueh H.C., Churchill S., Kohane I. (2010). Serving the enterprise and beyond with informatics for integrating biology and the bedside (i2b2). J. Am. Med. Inform. Assoc..

[10] Canuel V., Rance B., Avillach P., Degoulet P., Burgun A. (2015). Translational research platforms integrating clinical and omics data: A review of publicly available solutions. Brief. Bioinform..

[11] Vaught J., Lockhart N.C. (2012). The evolution of biobanking best practices. Clin. Chim. Acta.

[12] Genomics Research & Innovation Network (GRIN). https://www.grinnetwork.org/.

[13] Karlson E.W., Boutin N.T., Hoffnagle A.G., Allen N.L. (2016). Building the Partners HealthCare Biobank at Partners Personalized Medicine: Informed Consent, Return of Research Results, Recruitment Lessons and Operational Considerations. J. Pers. Med..

[14] Boutin N.T., Mathieu K., Hoffnagle A.G., Allen N.L., Castro V.M., Morash M., O’Rourke P.P., Hohmann E.L., Herring N., Bry L. (2016). Implementation of electronic consent at a Biobank: An opportunity for precision medicine research. J. Pers. Med..

[15] The Office of the National Coordinator for Health Information Technology (2016). Adoption of Electronic Health Record Systems among U.S. Non-Federal Acute Care Hospitals: 2008–2015.

[16] Aschettino L., Baldwin K., Friedman B., Grady R., Grebner L., Hennings M.E., Kadlec L., Kirby A., Meyer M., O’Dell R.M. (2015). Including patient-generated health data in electronic health records. J. AHIMA.

[17] Wood W.A., Bennett A.V., Basch E. (2015). Emerging uses of patient generated health data in clinical research. Mol. Oncol..

[18] Mandl K.D., Bourgeois F.T. (2017). The Evolution of patient diagnosis: From art to digital data-driven science. J. Am. Med. Assoc..

[19] Marsolo K., Corsmo J., Barnes M.G., Pollick C., Chalfin J., Nix J., Smith C., Ganta R. (2012). Challenges in creating an opt-in biobank with a registrar-based consent process and a commercial EHR. J. Am. Med. Inform. Assoc..

[20] McGregor T.L., Van Driest S.L., Brothers K.B., Bowton E.A., Muglia L.J., Roden D.M. (2013). Inclusion of pediatric samples in an opt-out biorepository linking DNA to de-identified medical records: Pediatric BioVU. Clin. Pharmacol. Ther..

