# Supplementary material for: Development of the Precision Link Biobank at Boston Children’s Hospital: Challenges and Opportunities
Source: J Pers Med. 2017 Dec 15;7(4):21. doi: 10.3390/jpm7040021 (PMC5748633; doi:10.3390/jpm7040021)
Supplement: Supplementary file 1 [file jpm-07-00021-s001.pdf]

Protocol Title: Boston Children's Biobank for Health Discovery ("Biobank")

Why is this research study being conducted? What is its purpose?

The goal of this research project is to collect samples and health information from patients and families and store them in an institutional Biobank to support use in future studies by Children's researchers and their collaborators. Researchers will use the samples and health information to conduct research on a variety of diseases, conditions and disorders with the hopes of finding new ways to detect, treat, and maybe prevent or cure disease. Some research may also lead to new products, such as drugs or tests for diseases.

This research project involves the storage and use of samples (such as blood and tissue) that are collected from patients as part of their routine medical care. We would like to collect these left-over clinical samples that might otherwise be discarded.

This study may store samples or may extract and store different parts of those samples, such as whole cells, or DNA, RNA, and proteins from those cells. All living things are made of cells. DNA is found in cells and contain genetic information or "genes". Genes contain instructions which tell our bodies how to grow and work and determine physical characteristics, such as hair and eye color. Small differences in genes can sometimes affect what diseases people are at risk for and possibly how they respond to certain treatments.

Who is conducting this research study, and where is it being conducted?

Kenneth Mandl, MD, MPH, Director of the Computational Informatics Program, is in charge of this study. This study will be conducted at Boston Children's Hospital and its satellite locations.

How are individuals selected for this research study? How many will participate?

Any patient seen at Boston Children's Hospital and their family members may participate in the study regardless of the reason for their visit to the hospital. Because we hope to approach every patient and family visiting Boston Children's, we estimate we might enroll at least 2000 participants a year but the number could be higher based on interest in the project.

What will happen if I am in this research study?

What samples may be collected? If you participate, we may collect and store your left-over clinical samples from any completed or future clinical tests/procedures at Boston Children's Hospital. If you/your child have not or will not have any clinical tests or procedures at Boston Children's Hospital, there will not be any left-over samples for us to store in our Biobank.

You/your child also have the option to donate an extra tube of blood to the Biobank for this research project. An extra tube of blood would be drawn at the time of your/your child's next clinical draw at the hospital and would not require a separate visit. Alternatively, you/your child may choose to get this extra blood drawn solely for research even if you do not have a clinical draw scheduled. You will not be charged for this draw. We will not collect more than 1 tablespoon of blood from you/your child at any time for our research. This extra blood may be collected specifically to store whole blood or products from the blood like DNA, RNA, proteins, or cells. The extra blood draw ensures that a sample is stored in the Biobank, as you/your child may not have any left-over samples from clinical tests/procedures.

Optional Blood Draw:

___: I/My child agrees to have an extra tube of blood drawn for research purposes.

___: I/My child DO NOT agree to have an extra tube of blood drawn for research purposes.

What information is collected? At the time of enrollment, we will ask you for some basic demographic information and how to contact you. You can skip any questions that you would not like to answer. We will also obtain health information from your Boston Children's medical record. This could include, but is not limited to, information from tests (lab results, etc.), medical procedures (operative notes, etc.), or information from clinic visits (notes, growth charts, height and weight, etc.). We will continue to obtain updates on your health from your medical record from a few times a year up to a few times a month for as long as you participate in this study. This health information may be linked with the samples we collect for research purposes.

Will I be contacted in the future? We may contact you if the Biobank study changes or if there are study updates that are important for you to know. If consent is being provided by a parent or legal guardian, we will contact the participant through email and/or mail when he or she turns 18 years old to remind him or her of their enrollment and provide them with the opportunity to re-enroll as an adult or withdraw from the study. In addition, Biobank staff may re-contact you for additional data and/or participation in clinical studies**.** By participating in the Biobank, you allow the Biobank staff to contact you to: 1) ask you for more information about your health, 2) donate additional samples, and 3) ask if you are interested in joining other research studies. All requests for more information, samples, and potential enrollment in other studies, are optional and will not affect your participation in the Boston Children's Biobank.

What happens to the samples and information? The samples (blood, tissue, etc.) and health data will be stored in the Boston Children's Biobank with some of your identifying information including, but not limited to, name, medical record number (MRN), and date of birth. The Biobank laboratory and study staff use this information to track your samples more effectively. The Biobank is intended to serve the BCH research community for years, so there is no limit to the length of time we will continue to keep your samples and health data. The samples and data are stored until requested by researchers as outlined below.

What research can be done? Researchers may use samples and health data for many types of medical research, including biological and genetic research. Your samples, and parts of these samples (such as DNA and RNA from blood or tissue), and health data can be used for research on any disease or disorder. You may learn what research studies are using the Biobank, however you cannot decide when your samples and health information are used in a study.

Research may include but is not limited to:

- We may study any of your samples under the microscope to better understand biological processes in healthy individuals and people with disease.
- We may create a "cell line" from your sample. This involves collecting cells (the building blocks of our tissues/body) from your sample and treating them so they grow in a dish. "Cell lines" provide an unlimited supply of cells for research and can model various health conditions.
- We may use your cells to create pluripotent stem cells. This type of cell can be used to create different types of tissue, such as heart or muscle cells. Your cells might be used in research that alters genes in the cells to study different diseases and normal biological processes.
- Researchers may perform genetic analyses, including whole genome analysis or whole genome/exome sequencing, on your DNA sample. Sometimes researchers study just a few areas of your genetic code that are linked to a disease or condition. In whole genome studies or sequencing, all or most of your genes may be analyzed and used by researchers to study links to many diseases or conditions.

Who may see, use or share your health information and samples?

- Boston Children's researchers and their collaborators may search a limited set of information that is stored

in the Biobank to decide if it would meet their research needs. This "summary" information does not include your name or any other information that would directly identify you.

- Your samples and health information may be used by many researchers for different types of studies. Most researchers do not need any information that could identify you (such as name, address, etc). These researchers may have access to your samples and health information after obtaining approval from the Biobank Scientific Review Committee.
- Only Boston Children's researchers may request identifying information. Any BCH researcher who requests identifying information from the Biobank must first get approval from the BCH Institutional Review Board (IRB) and also approval from the Biobank Scientific Review Committee. Researchers may need your identifying information to do a more in-depth review of your medical record to get health information important for their specific research study.
- Some researchers who use the Biobank may collaborate, or work with, other institutions such as other hospitals, universities, businesses that conduct research, etc. Any samples and health information that are shared with other institutions will be coded with IDs so they cannot be directly linked to you.

Your samples, genetic data from your samples (e.g. DNA sequence), and health information may be shared with central repositories' within Boston Children's and external repositories such as those sponsored by the National Institutes of Health (an agency of the federal government). Your name and other direct identifying information will not be included. These repositories allow researchers to share information for studies that would otherwise not be possible. Researchers who want to use samples or information in external repositories must apply to those repositories before any samples and information are shared.

Is any information put in the medical record? Since this is a hospital-wide project, we may use some of the hospital systems, including the medical record system, to record your participation decision or to remind us to ask you if you want to participate. Your decision to participate will be marked in your medical record. If you choose to donate an extra tube of blood, the order for the research blood draw will be in the medical record.

A copy of this consent form will be placed in your medical record. If you do not have a medical record at Boston Children's Hospital, one will be created for you.

Will I get results of research done on my samples?

You should not expect to get individual results from research done through the Biobank. It is possible that your samples and health information may never be used in a research project. We will not give any research results about you to your doctor or put them in your medical record. However, there is a small chance that researchers could discover something that might be urgently important to your health or medical care. If this happens, we will contact you to see if you want to learn more.

What are the risks of this research study? What could go wrong?

Risk of Sample Collection: There are few risks associated with participation since most of the sample collection for this study is from samples already obtained for clinical purposes. If you choose to donate an extra tube of blood for research, the risks associated with a blood draw are minor discomfort and bruising.

Risk of Sample and Information Storage: We will store your samples in one research bank and your medical information in another research bank. Researchers will be able to search across both of the research banks. We will try to keep the information secure and private by storing all of the data in password protected, secured databases. Only approved researchers and study staff will have access to parts of the information. However,

even with these protections there is a risk that someone could get access to the data we have stored about you, breaching your confidentiality.

Risk of Genetic Research: It is possible this type of research may uncover information about your genes that may put you or a relative at risk for a genetic disorder in the future. Genetic information divulged to the wrong source could affect you and your family if an insurance company or employer acquired this genetic information. This is unlikely since there are federal and state laws in place that help prevent discrimination based on genetics. Also, the Biobank will do its best to keep all information confidential.

To help to lessen these risks, we have a Certificate of Confidentiality (CC) from the US government. It protects your ability to remain confidential in a research project by giving us the right not to identify you, even under a court order or subpoena. Still, the Biobank may report medical information (if you need medical help), probable harm to yourself or others, or probable child abuse or neglect, and the government may see your information if it audits us. The CC does not prevent you or a member of your family voluntarily releasing information about your involvement in research. If an insurer, employer, or other person obtains your written consent to receive research information, then the Biobank may not use the Certificate to withhold that information.

What are the benefits of this research study?

You will not directly benefit from being in this study. We hope that research from studies using the Biobank will allow us to better understand factors that impact health. This research may improve how doctors prevent, diagnose, and treat diseases in the future.

Are there costs associated with this research study? Will I receive any payments?

There are no costs to you or your insurance. You will not be paid for joining the study. Research approved by the Biobank may lead to new products, research tools, or inventions that are patented. If these lead to payment to Children's Hospital, the payments will be devoted to supporting research and providing health care. Participants will not receive any portion of those payments.

If I do not want to take part in this research study, what are the other choices?

Participation in this research is voluntary and you should not feel any pressure to participate. If you do not want to participate, it will not interfere with any current or future care you or your family receives at Boston Children's and there will be no penalty or loss of benefits**.** If you decline to enroll in this study, your/your child's clinical samples will either be maintained or discarded as per the current clinical policies of the hospital. If you are unsure if you would like to participate, we will register you as undecided and may approach you in the future about participation.

What are my rights as a research participant? May I withdraw?

You may withdraw from the study at any time by calling the Biobank (see pg. x for contact information) and a member of our study staff will be able to assist you. If you/your child choose(s) to withdraw, your/your child's data and samples will no longer be distributed for research purposes though some information may remain in the Biobank for internal auditing purposes only. Any data or samples already given to researchers prior to your decision to withdraw will remain as a part of research until that study is completed.

Your privacy rights:

You/your child's health information is protected by a law called the Health Information Portability and Accountability Act (HIPAA). In order to create and manage the information in a repository it is necessary to allow for certain people to see information collected for this research project that may include information about you. The only people who will see identifying information about you are approved members of the Biobank

staff, Boston Children's research operations, BCH researchers that utilize Biobank data, and agencies, sponsors or companies that may support research operations with appropriate approval. Also, if some law or court requires us to share the information, we would have to follow that law or final ruling.

Because research using Biobank data and samples is ongoing, we cannot give you an exact time when we will destroy your information. Results from this project may be published in a medical book or journal or be used for teaching purposes. However, your name or identifying information will not be used without your specific permission. You/your child may have the right to get some of the information that was shared with others for research, treatment or payment. This information is available after the study analysis is done. To request the information, please contact the Hospital's Privacy Officer at xxx-xxx-xxxx.

If you/your child decide to share private information with anyone not involved in the study, the federal law designed to protect privacy may no longer apply to this information. Other laws may or may not protect sharing of private health information. If you have a question about this you may contact the Boston Children's Hospital Privacy Office at xxx-xxx-xxxx, which is set up to help you understand privacy and confidentiality.

DATA USE AGREEMENT (De-Identified or Limited Data Set)

This Data Use Agreement ("Agreement"), effective as of the ______________ ("Effective Date"), is made by and between The Children's Hospital Corporation d/b/a Boston Children's Hospital ("Provider"), located at 300 Longwood Avenue, Boston, MA 02115, and _________________located at _________________("Recipient"). Provider and Recipient are referred to in this Agreement individually as a "Party" and collectively, as the "Parties".

Whereas, the Parties wish to enter into this Agreement so that Provider may share with Recipient certain protected health information ("Data") for the purpose of research and in a manner that complies with federal, state and local laws, including 45 C.F.R. 164 ("HIPAA").

Now therefore, the Parties hereby agree as follows:

1. The "Data"[1] transferred to Recipient under this Agreement is: _____________________________
____
____

2. Recipient may use and/or disclose the Data for its Research and Public Health activities under Protocol Boston Children's Biobank for Health Discovery ("Study").

3. The Recipient shall conduct the Study under the direction of ___________ ("Recipient Scientist").

4. Provider retains ownership of the Data, which includes any related know-how provided to Recipient along with the Data.

5. Recipient agrees to use, store, and disclose the Data solely for the performance of the Study, any subsequently agreed upon purposes, and as required by law.
   5.1. Recipient shall conduct the Study in compliance with all applicable federal, state and local laws, rules and regulations including, but not limited to laws regarding the privacy or protection of personal or medical information, and HIPAA.

6. Recipient agrees to use appropriate safeguards to prevent any use or disclosure of the Data other than as specified in this Agreement or required by law.

DO NOT COPY

[1] Data shall not include any of the following "Prohibited Identifiers": names, postal address information other than towns, cities, states and zip codes, telephone and fax numbers, e-mail addresses, URLs and IP addresses, social security numbers; medical record numbers; health plan beneficiary numbers; account numbers; certificate and license numbers; vehicle identification numbers; device identifiers and serial numbers; biometric identifies (such as voice prints and finger prints); and full face photographs or comparable images. If any of the Prohibited Identifiers are included in the Data, a Business Associate Agreement must be executed and attached as an exhibit to this Agreement.

© Boston Children's Hospital, 2011 All rights reserved

DO NOT COPY

6.1. Recipient will not use the Data, alone or in combination with other information, to identify the individuals or contact the individuals from whom the Data was derived.

7. Recipient agrees that the Data: (a) is to be used solely for teaching and academic research purposes; (b) is to be used only at Recipient, by Recipient Scientist or under the direction of the Recipient Scientist; (c) will not be transferred to anyone else at Recipient or to any other third party without Provider agreement. The Recipient will ensure that any agent, including a subcontractor, to whom the Recipient provides the Data received from Provider, agrees to the same restrictions and conditions that apply through this Agreement to Recipient with respect to such information.
   7.1. The Recipient will promptly report in writing to Provider any use or disclosure of the Data not provided for by this Agreement of which the Recipient becomes aware.

8. Provider agrees to notify Recipient of any restriction to the use or disclosure of the Data that Provider has agreed-to with an individual, in accordance with HIPAA to the extent that such restriction would affect Recipient's use or disclosure of Protected Health Information.

9. The Parties acknowledge that the Study is a collaborative effort. The Parties agree to coordinate their respective activities regarding publication prior to submission of a paper or abstract for publication. The purpose of this coordination is to ensure the proper collation and presentation of Data and to reflect the collaborative nature of the Study.
   9.1. In the event of publication or disclosure of results that is not a joint publication or disclosure, the publishing Party shall grant the other party the opportunity to review and/or comment on such proposed publication, abstract, or oral presentation. The publishing Party shall grant the non-publishing party no less than seven (7) days to review such proposed disclosure. The non-publishing Party may reasonably request in writing that the proposed publication or disclosure be delayed for up to an additional thirty (30) days as necessary for the filing of a patent application. The non-publishing Party may further request that its confidential information be deleted, but at no time will the publishing Party be required to remove any information relating to the results of the Study, or any other information that is reasonably required by the publishing source to be included in the publication or presentation.
   9.2. The publishing Party agrees that the source of the Data shall be acknowledged in accordance with scientific custom in all published or oral communications concerning the Study.

10. Recipient agrees to indemnify Provider for any liability, loss, claim, or demand arising from its use, storage or disclosure of the Data. Recipient assumes all liability for damages which may arise from Recipient's unauthorized use, storage or disclosure of the Data.

11. Recipient is responsible for the safe handling and destruction or return (including the cost of the return) of the Data in accordance with all federal and state laws, and in the manner agreed to by the parties.

© Boston Children's Hospital, 2011 All rights reserved

12. Recipient agrees that the Data provided is experimental in nature, and Provider makes no warranties, expressed or implied, regarding the quality of any product produced under this Agreement.

13. This Agreement shall remain valid for so long as Recipient retains the Data, unless sooner terminated as set forth in this Agreement.
    13.1. Recipient may terminate this Agreement at any time by notifying Provider and returning or destroying the Data.
    13.2. Provider may terminate this Agreement at any time, for any reason, by providing 30 days' prior written notice to Recipient.
    13.3. Provider will provide written notice to Recipient within 10 days of any determination that Recipient has breached a material term of this Agreement. Provider will afford Recipient an opportunity to cure said alleged material breach upon mutually agreeable terms. Failure to agree on mutually agreeable terms for cure within 30 days will be grounds for the immediate termination of this Agreement by Provider.
    13.4. Except as provided in this Section 13, upon termination of this Agreement, for any reason, Recipient shall return or destroy all Data received from Provider, created or received by Recipient on behalf of Provider. This provision shall apply to Data that is in the possession of subcontractors or agents of Recipient.
    13.5. Recipient shall retain no copies of the Data. In the event that Recipient determines that returning or destroying the Data is infeasible, Recipient shall extend the protections of this Agreement to such Data and limit further uses and disclosures of such Data to only those purposes that make the return or destruction infeasible, for so long as Recipient maintains such Data.

14. Sections 2, 4, 5, 6, 7, 8, 9, and 13.1, 13.2, 13.3 of this Agreement will survive any termination of this Agreement.

15. Recipient will report any violation to this Agreement to Boston Children's Hospital's Clinical Trials Business Office.

16. This Agreement shall be governed by and interpreted under the laws of the Commonwealth of Massachusetts.

17. This document states the entire agreement between the parties regarding De-Identified or Limited Data Sets provided by Provider to Recipient. It may not be amended or modified except through a later written agreement, signed by both parties, and expressly-referencing this Agreement.

DO NOT COPY

[signatures to follow]

© Boston Children's Hospital, 2011 All rights reserved
